# Cold Atmospheric Plasma Induces a Predominantly Necrotic Cell Death via the Microenvironment

**DOI:** 10.1371/journal.pone.0133120

**Published:** 2015-08-14

**Authors:** François Virard, Sarah Cousty, Jean-Pierre Cambus, Alexis Valentin, Philippe Kémoun, Franck Clément

**Affiliations:** 1 Centre de Recherche en Cancérologie de Lyon, UMR5286 CNRS/INSERM/Université Lyon 1 UCBL, Lyon, France; 2 Faculté d’Odontologie de Lyon, Université Lyon 1, Lyon, France; 3 Université Paul-Sabatier Toulouse III, Faculté de Chirurgie Dentaire de Toulouse, Toulouse, France; 4 CHU de Toulouse, Toulouse, France; 5 Laboratoire Hématologie, Hôpital Rangueil, TSA 50032, Toulouse, France; 6 Université Paul-Sabatier Toulouse III, Faculté de Pharmacie UMR 152 IRD-UPS PHARMA-DEV, Toulouse, France; 7 LCABIE—IPREM / UMR 5254, Université de Pau et des Pays de l'Adour Pau, Pau, France; University of Pecs Medical School, HUNGARY

## Abstract

**Introduction:**

Cold plasma is a partially ionized gas generated by an electric field at atmospheric pressure that was initially used in medicine for decontamination and sterilization of inert surfaces. There is currently growing interest in using cold plasma for more direct medical applications, mainly due to the possibility of tuning it to obtain selective biological effects in absence of toxicity for surrounding normal tissues,. While the therapeutic potential of cold plasma in chronic wound, blood coagulation, and cancer treatment is beginning to be documented, information on plasma/cell interaction is so far limited and controversial.

**Methods and Results:**

Using normal primary human fibroblast cultures isolated from oral tissue, we sought to decipher the effects on cell behavior of a proprietary cold plasma device generating guided ionization waves carried by helium. In this model, cold plasma treatment induces a predominantly necrotic cell death. Interestingly, death is not triggered by a direct interaction of the cold plasma with cells, but rather via a transient modification in the microenvironment. We show that modification of the microenvironment redox status suppresses treatment toxicity and protects cells from death. Moreover, necrosis is not accidental and seems to be an active response to an environmental cue, as its execution can be inhibited to rescue cells.

**Conclusion:**

These observations will need to be taken into account when studying *in vitro* plasma/cell interaction and may have implications for the design and future evaluation of the efficacy and safety of this new treatment strategy.

## Introduction

Plasma medicine is an emerging therapeutic field based on the use of cold and partially ionised gases produced by various processes at atmospheric pressure. Among the technologies developed, one Cold Atmospheric Plasma (CAP) category consists in the production of ionization waves in the air, currently called in the literature “plasma jets”, and producing numerous reactive species [1–13]. Other terminologies have been proposed based on physical properties, such as Pulsed Atmospheric Pulsed Stream (PAPS) [14], Guided Streamers (GS) [15,16], and Guided Ionization Waves (GIW) [17].

Several studies suggest that these technologies may be useful in sterilization, blood coagulation, wound healing, or cancer treatment. Key advantages of CAPs are that they could be tuned to obtain different biological effects in absence of toxicity for normal adjacent tissues [18]. However, data on plasma mechanisms of action at the cellular level are rather scarce, as *in vitro* plasmas/cell interactions can be challenging to interpret due to variable, and sometimes contradictory, outcomes.

We decided to study the interaction of GIW carried by Helium (He-GIW) with a normal human fibroblast population isolated from periodontal ligament (hPDL) [19]. PDL is a specialized connective tissue that participates in anchoring the teeth and is destroyed during periodontitis. Currently, the prognosis of periodontitis is unpredictable and attempts to regenerate tooth anchorage in order to prevent its loss continue to be disappointing [20,21]. CAP is being considered as a potential therapeutic option for this unmet medical need.

Pleiotropic effects of CAP on mammalian cells have been reported, ranging from disturbing cell adhesion to cell death induction [22]. Cell death can be triggered by harsh physical conditions that disrupt vital cellular functions, a process regarded as passive and accidental. It can also occur and be executed in a programmed way whereby it becomes an essential part of development, homeostasis, wound healing, or pathological processes [23]. Apoptosis, the prototypical controlled cell death, is based on energy-dependent self-destruction with cytoplasm shrinkage, nuclear condensation, and plasma membrane blebbing, with prolonged plasma cell integrity. On the other hand, necrosis has been considered for a long time as a non-specific and uncontrolled form of cell death, with rapid loss of cellular membrane potential resulting in cytoplasmic swelling and rupture of the plasma membrane. However, accumulating evidence suggests that some forms of necrosis are induced in a specific and controlled way, renewing the interest for this cell death mechanism [24–26].

All forms of controlled cell deaths occur through the same sequence of events: trigger, initiator, mediator, and executioner. For example, apoptosis is triggered by death receptor activation (extrinsic) or by mitochondrial cytochrome C release (intrinsic) and is executed by caspase 3/7. On the other hand, controlled necrosis can be triggered by structurally unrelated stimuli (ROS, Ca^2+^, Hypoxia, receptor engagement, etc…) and its execution relies on ATP depletion, osmotic swelling, and/or loss of lysosomal integrity. In contrast to accidental cell death, progression through the different layers of controlled cell death can be inhibited. Inhibiting cell death execution can result either in cell survival or cell death by another pathway due to the interconnection between cell death programs [25,26].

In this paper, we describe the induction of controlled necrosis in human normal hPDL cells treated with a He-GIW atmospheric cold plasma. We show that cell death is triggered by transient changes in the cell’s environment and not by direct plasma-cell interaction, and that changing the microenvironment’s redox-status can protect cells from cell death. Moreover, necrosis seems to be an active response to an environmental cue, since it can be inhibited to rescue cells.

## Materials and Methods

### Helium Guided Ionization Waves (He-GIW) device

**Fig 1:**
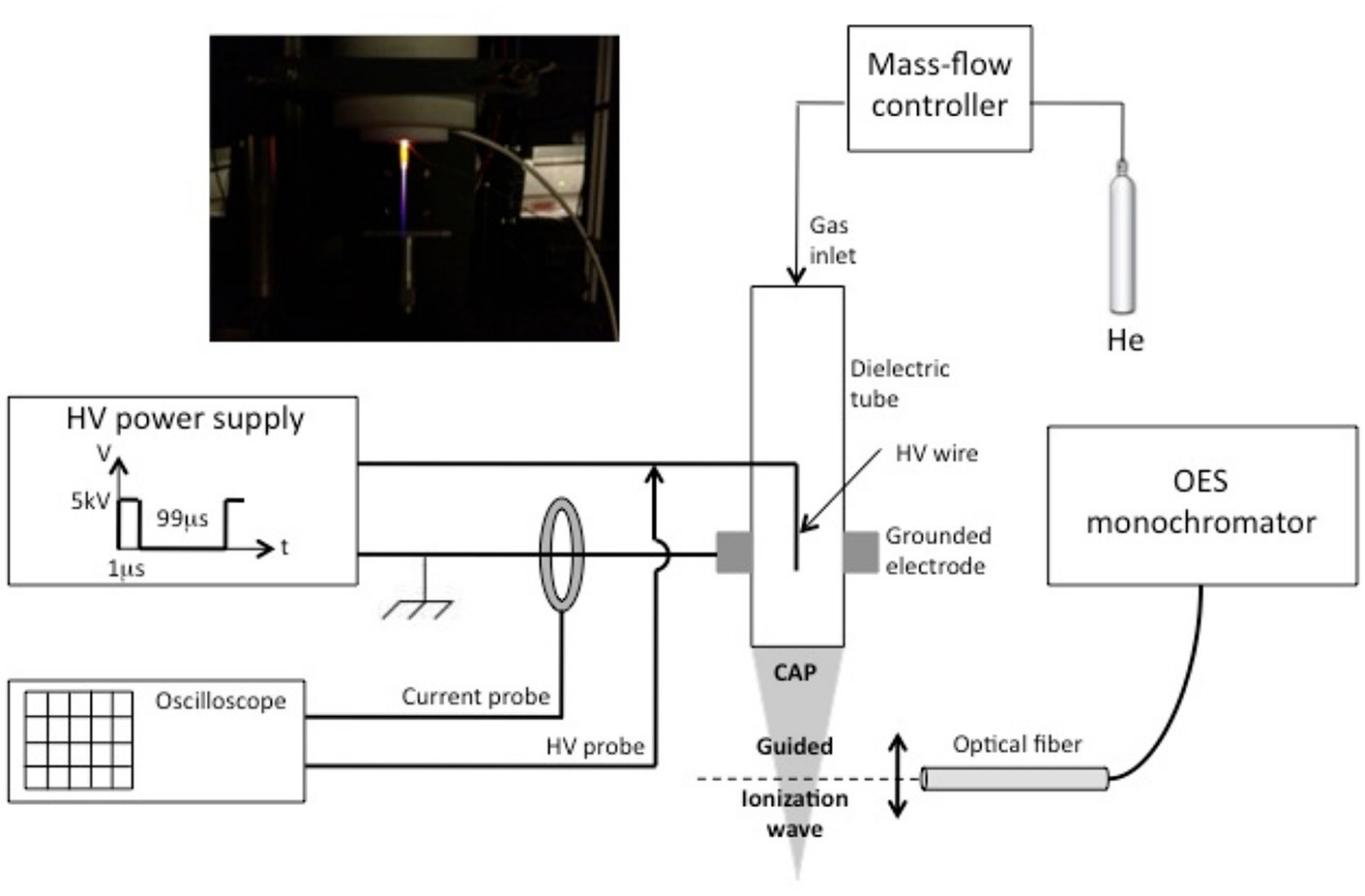
Experimental set-up.

The plasma process consists in the production of Guided Ionization Waves at atmospheric pressure and room temperature. This device is composed of three major elements: the reactor in which gas ionization is initially produced, the High-Voltage generator which supplies sufficient electrical energy in the gas for its ionization, and finally the gas used and its hydrodynamic conditions of flow in the reactor. A description of this device is indicated in Fig 1.

The reactor is a dielectric tube in alumina in which a tungsten filament is inserted and high-voltage powered. A metallic cylinder is fixed around the dielectric tube and grounded, thus allowing the application of high electric fields between the tungsten filament and this metallic cylinder. This configuration allows limiting the current and avoids the formation of electrical arcs. Due to the very thin diameter of the tungsten filament, a point effect induces the formation of specific ionization waves, which are guided by the dielectric tube and propagate in the surrounding air until several centimeters.

**Fig 2:**
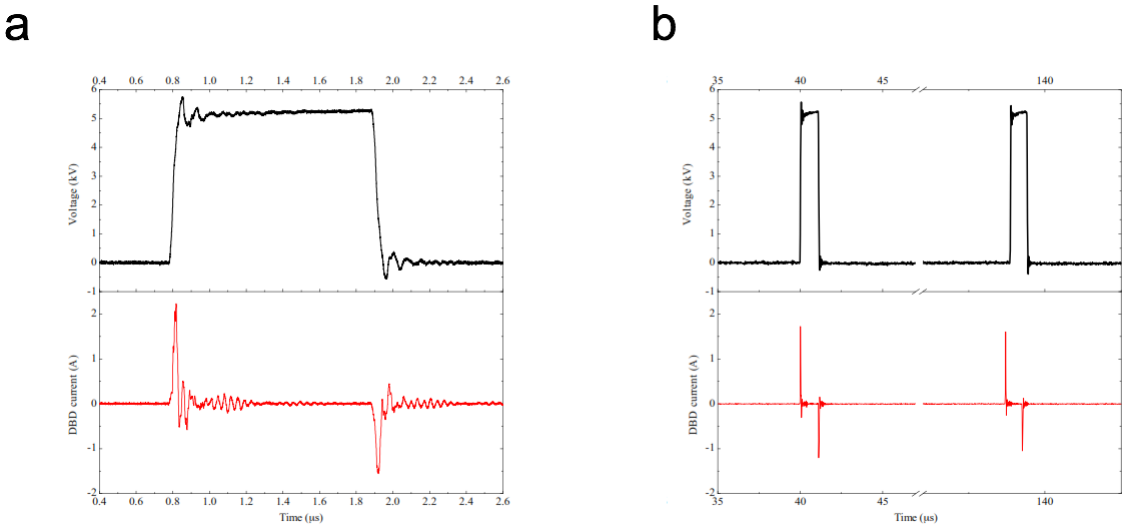
Electrical high voltage and current signals analyzed during one impulse (2a) and during one period (2b) (He gas flow = 2slm, voltage frequency = 10kHz, voltage amplitude = 5kV, voltage duty cycle = 1).

The High-Voltage power supply delivers periodic high-voltage pulses of short durations (1μs) and low frequency (~10 kHz), so that the pulse duration is largely shorter than the period of the signal. Duty cycle of the voltage signal is thus fixed to 1%, rise and fall times of this delivered signal are closed to 100ns each other. Electrical characterization is performed using specific High Voltage and current probes (Tektronix P6015 HV 1000:1 probe—bandwidth 75MHz, and current probe Tektronix CT2—bandwidth from 1.2kHz to 200MHz). Measurements are registered with a Tektronix oscilloscope (TDS3054B, 500MHz– 5GS/s) as indicated in Fig 2a, where it can be observed that substantial displacement currents produced. These currents are positive (~2,1A peak) during the rise time and negative (~-1,6A peak) with the decrease and are separated by less than 100ns each other (Fig 2b). The system thus produces pulsed GIWs, which considerably limits the gas temperature that remain close to room temperature [27,28] Estimates of the pulsed power injected into the gas by integration of the current signal during the application of the voltage are in the kilo-Watt range and the energy is in the low milli-Joules range, which is in accordance with measurements on devices with similar configuration [29].

Helium gas was chosen for its low breakdown voltage and because it generates a better propagation of ionization waves, with a flow at a constant rate of 2 standard liters per minutes (slm) inside the ceramic tube. Once gas breakdown has been achieved, the formation of a laminar gaseous plasma pencil is easily visible as indicated in Fig 1. The colors observed are due to the emissive species produced by the interaction of the ionization wave with the surrounding air, leading to the excitation, dissociation, and ionization of gaseous molecules. Identification of specific emissive species has been performed using optical emission spectroscopy (OES) and has been recently been reported for several gaseous mixtures of Helium or Argon with Nitrogen or Oxygen [28]. Main emissive species with Helium as plasma carrier gas are listed in table 1, highlighting that the production of Reactive Oxygen and Nitrogen Species (ROS/RNS) in the gas phase are mainly NOγ system, radical hydroxyl OH, atomic oxygen or specific neutral and ionized molecular nitrogen species.

**Table 1:** Main emissive species produced with Helium as plasma carrier gas.

Species	λ(nm)
NOγ: A^2^Σ^+^−X^2^Π	vibrational transitions in the 200-280nm range
OH: A^2^Σ^+^−X^2^Π	309
N_2_(SPS): C^3^Π_*u*_−B^3^Π_*g*_	315,93 / 337,13 / 357,69 / 380,49
N_2_^+^(FNS): B^2^Σ_*u*_^+^−X^2^Σ_*g*_^+^	391,44 / 427,81 / 470,92
N_2_(FPS): B^3^Π_*g*_−A^3^Σ_*u*_^+^	transitions in the 500-900nm range
Helium transitions	500-750nm range
H_α_: n = 3-n = 2	656,30
O: ^5^P-^5^S^0^ / ^3^P-^3^S^0^	777,53 / 844,63

**Fig 3:**
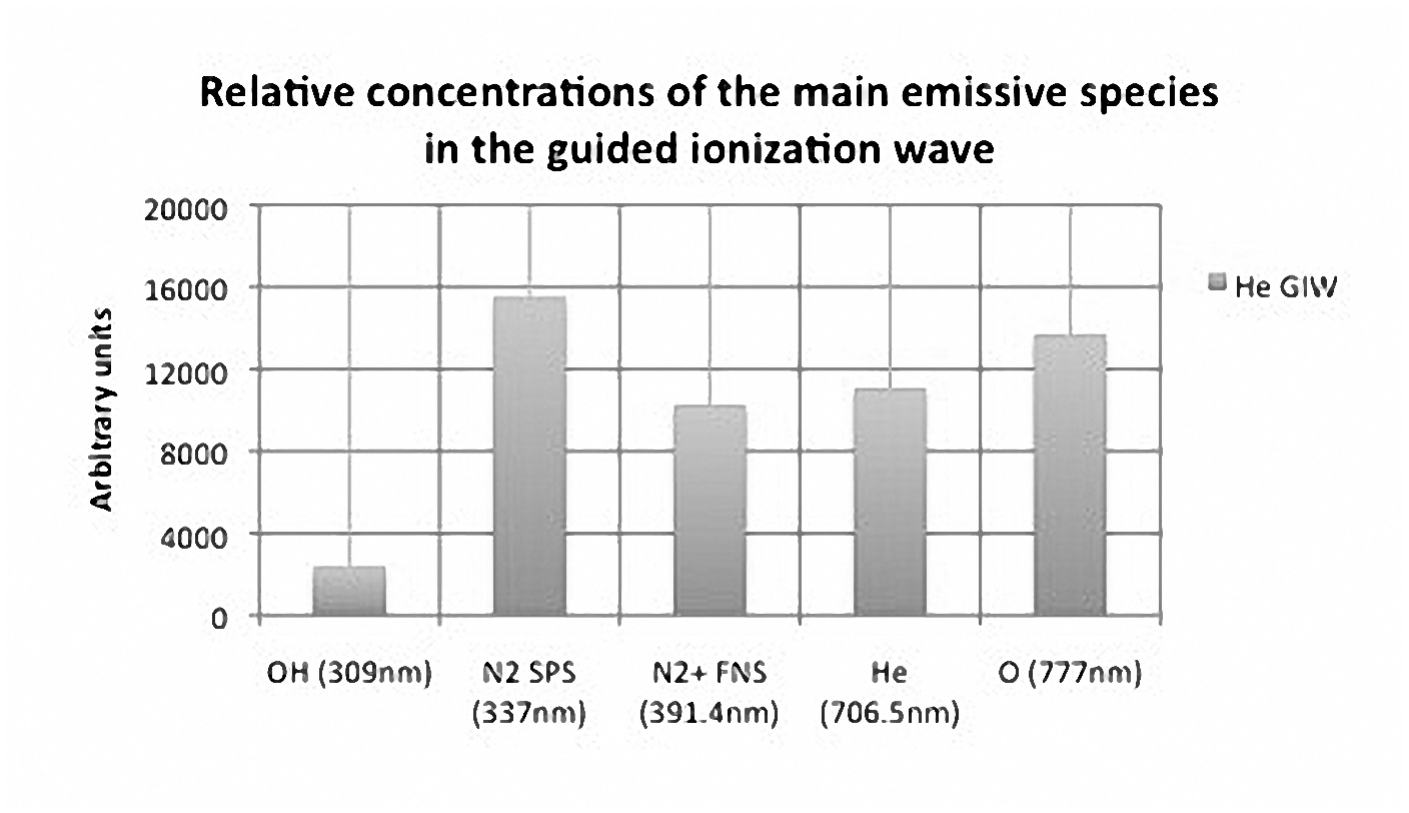
Relative concentration of the main emissive species in the guided ionization wave (conditions: He gas flow = 2slm; voltage frequency = 10kH; voltage amplitude = 5kV; voltage duty cycle = 1%. Optical fiber placed 5mm after the exit ceramic tube).

The relative intensities of the main emissive species in the guided ionization wave were performed by optical characterizations 5mm after the exit ceramic tube along the axial propagation of plasma (Fig 3).

The gas temperature of our device was previously estimated by rotational temperatures of probes molecular as hydroxyl OH and Second Positive System N2 [28]. The temperature obtained 5mm at the ceramic tube exit are closed to 315K. Because the temperature of the plasma ionization wave is decreasing along its propagation (temperature gradient) to reach ambient air temperature, our plasma process (used at 30mm from the cell culture medium surface) does not interfere the cell viability by thermal changes.

### Reagents

Propidium iodide (PI), 7-aminoactinomycin D (7-AAD), Annexin V Apoptosis Detection Kit APC and isotype-matched negative control antibody were from Ebioscience (Paris, France). The Pan Caspase Inhibitor Z-VAD-FMK (1/1000) was from R and D system (Minneapolis, MN, USA). N-acetyl-L-cysteine (NAC), were purchased form Sigma Aldrich, St Louis, MO, USA). Rabbit anti human antibody specific for the cleaved (active) form of caspase-3, 3,3'-Dihexyloxacarbocyanine Iodide DiOC 6(3), Dylight 647 goat anti-rabbit IgG, Hoechst 33342, Accutase, penicillin, streptomycin, phosphate buffer saline, α and D Minimum Essential Medium (MEM) Glutamax, fetal calf serum and goat serum, were from Invitrogen (Carlsbad, CA, USA). Rabbit polyclonal anti-caspase-3 antibody was from Cell Signaling Technology (Danvers, MA, USA).

### Human PDL cells isolation and cell culture

Human PDL (hPDL) cells were isolated from non-impacted premolars extracted from healthy donors for orthodontic reasons (one female, five males; age range 13–18 years) and pooled. Briefly, PDL tissue was separated from the surface of the mid-third of the root and cells were recovered and cultured in growth medium (α-MEM Glutamax + 10% Fetal Calf Serum [FCS]) in 5% CO_2_ atmosphere as previously described [19]. Cells from passage 4 to 10 were used and all exhibited a CD105+/90+/45-/14- phenotype that confirmed their connective tissue lineage as well as the lack of cells from hematopoietic origin in the culture (data not shown). To test the effect of He-GIW on hPDL cultures, cells were seeded in 12- or 48-well plates in growth medium at different densities as indicated and allowed to attach for 24 h. Cells were then treated as described below.

The use of human waste tissue conforms to standard practices established at the University of Toulouse and regulated by the French Ministry of Health. Written informed consent was obtained from the patients’ parents. A declaration of human biological sampling (without cell collection freezing bank) was filed with the local ethics committee (CPP Sud-Ouest Outre-Mer).

### He-GIW treatment

One hour prior the test, culture medium was replaced by selected medium (1 mL and 0.25 mL for 12- or 48- well plates, respectively) supplemented or not with agonists or inhibitors as indicated, then direct He-GIW treatment was performed in open air, 30 mm above the bottom of each well. Preliminary experiments showed no difference in cell behavior between gas flow-exposed cultures and unexposed cultures, qualified in this study as control cells. Cells were then analyzed at indicated times as described below.

### Reactive oxygen species (ROS) inhibition

To inhibit ROS, cell cultures were pretreated with either NAC (4 mM) or 10% FCS for 30 min prior to treatment, or added to wells after exposure without medium change, except where indicated.

### Determination of cell apoptosis 6 and 24 h after He-GIW treatment

Untreated, He-GIW- or staurosporin-treated cells were washed with PBS then detached with Accutase. Single-suspensions of hPDL cells in annexin V buffer were pelleted at 500 rpm for 5 min before incubation with annexin V-APC conjugate (1/50; v/v) at RT for 30 min. Annexin V buffer was then added to cells without washing. Cell number was determined during FACS experiment using FluoSpheres Polystyrene Microspheres, 15 μm (Invitrogen). 7-aminoactinomycin D (7-AAD) or propidium iodide (PI) were added for cell death exclusion. Acquisition of events was performed on an LSR-2 or a FACScalibur cytometer (BD Biosciences, Franklin Lakes, USA). The data were analyzed with the FlowJo software (TreeStarinc., Ashland, USA).

### Western Blotting

Cells were lysed in cold lysis buffer (20 mM Tris-HCl (pH 7.5), 150 mM NaCl, 1% Nonidet NP40) supplemented with 1mM orthovanadate, 10mM NaF, and a protease inhibitor cocktail (Sigma-Aldrich) for 10 on ice. Cell lysates were cleared by centrifugation (16 000 g, 10 min, 4°C), and protein concentration was determined by the Bradford assay (Bio-Rad, Hercules, CA, USA). Proteins were resolved on SDS- PAGE, transferred onto PVDF membranes by electroblotting, and nonspecific binding sites were blocked using Tris-buffered saline containing 0.1% Tween-20 and 5% (w/v) dry milk or BSA. Primary antibody was incubated overnight at 4°C and after incubation with appropriate secondary antibodies conjugated to horseradish peroxidase, blots were revealed using the ECL reagents (GE Healthcare, Chalfont St. Giles, UK).

### Determination of mitochondrial membrane potential

Mitochondrial membrane potentials (Δψm) were measured by means of DiOC6 staining as previously described [30]. Briefly, untreated or He-GIW, staurosporin-treated cells were washed with PBS then detached with Accutase. Single-suspensions of hPDL cells in FACS buffer (PBS/ 5%FCS) were pelleted at 500 rpm for 5 min before incubation for 20 min in 350μl of 40 nmol/l DiOC 6(3) in FACS buffer, immediately followed by analysis on a cytofluorometer.

### Immunofluorescence studies

For fluorescence microscopy analysis, cells were seeded onto 48-wells, then subjected to He-GIW, staurosporin, or left untreated. Six hours later, cells were incubated with PI for 15 min, then fixed in 3.7% paraformaldehyde for 15 min, blocked in 5% goat serum for 30 min before incubation with an anti-cleaved caspase-3 primary antibody for 1 h before washing. Dylight 647 goat anti-rabbit IgG (Invitrogen) was used as secondary antibody. Nuclei were counterstained with Hoechst 33342 (1μg/mL), before final washes in PBS. Fluorescence staining was visualized by fluorescence microscopy (Zeiss Apotome).

### Statistical analysis

Data were expressed as mean ± SEM. Tests were performed in triplicate and each experiment was independently repeated at least three times. One-way and two-way analyses of variance were performed with Bonferroni correction for multiple comparisons. Statistical significance was chosen at 0.05 with p<0.05 (*), p<0.01 (**), and p<0.001 (***). Statistics and graphics were generated using PRISM GraphPad 5.0. (GraphPad Software, San Diego).

## Results

### Device settings and cell culture parameters influence cell fate after He-GIW exposure

**Fig 4:**
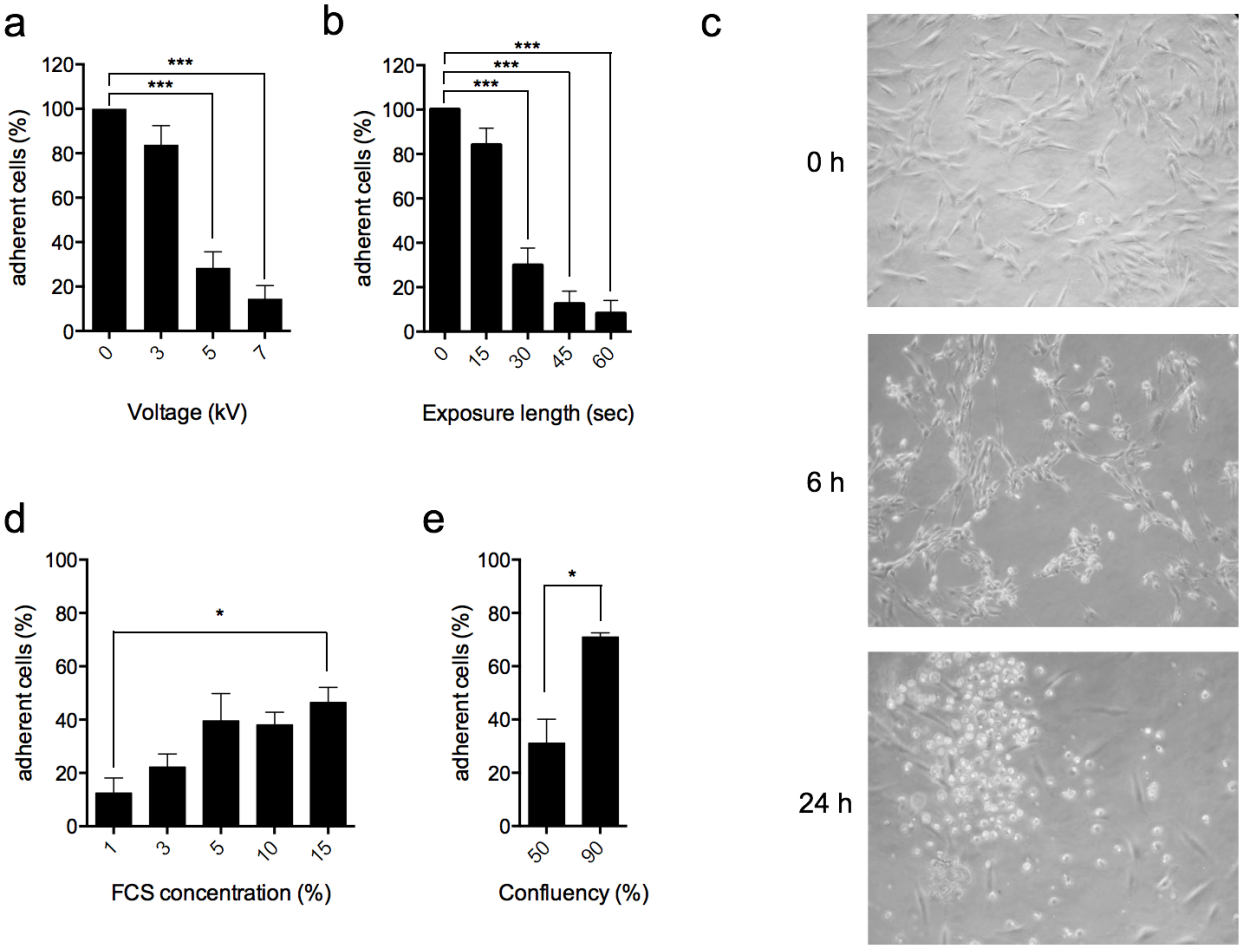
Device settings and cell culture parameters influence cell fate. **(a)** hPDL were treated with He-GIW using different voltages and **(b)** exposure lengths. Adherent cell number was evaluated 24h after treatment and expressed as a percentage of control. **(c)** Cell morphology was monitored by phase contrast microscopy 6h and 24h after treatment. **(d)** Cell culture incidence on He-GIW treatment was evaluated using different FCS concentrations and **(e)** cell confluence. Adherent cell number was assayed 24h after treatment and expressed as percentage of control. Error bars represent S.E.M. of three independent experiments p<0.05 (*), p<0.01 (**) and p<0.001 (***).

As a starting point, 50% hPDL confluent cells maintained in 3% FCS were treated with He-GIW at increasing voltage and exposure time. Twenty-four hours after treatment, we observed a significant decrease of adherent cell number with 5kV and 30 sec exposure, compared to control (Fig 4a and 4b). With these settings, the cell morphology shifted from normal elongated to refringent 6 h after treatment, and most cells floated in the medium culture 24 h after exposure (Fig 4c).

During preliminary experiments, we also observed that extracellular environment had a significant impact on cell fate after He-GIW treatment. Indeed, while exposed hPDL cultures maintained in 1% FCS exhibited a dramatic loss of adherent cells compared to control, increasing FCS concentration (Fig 4d) or confluence (Fig 4e) were able to protect cells against loss of adhesion. However, FCS protection was limited and reached a 50% cell recovery plateau starting at 5% FCS concentration (Fig 4d).

Based on these findings, we decided to focus next on the mechanisms inducing cell detachment using 5 kv and 30-second exposure length for plasma setting and 3% FCS, 50% confluence for cell culture.

### He-GIW induces a predominantly necrotic cell death

**Fig 5:**
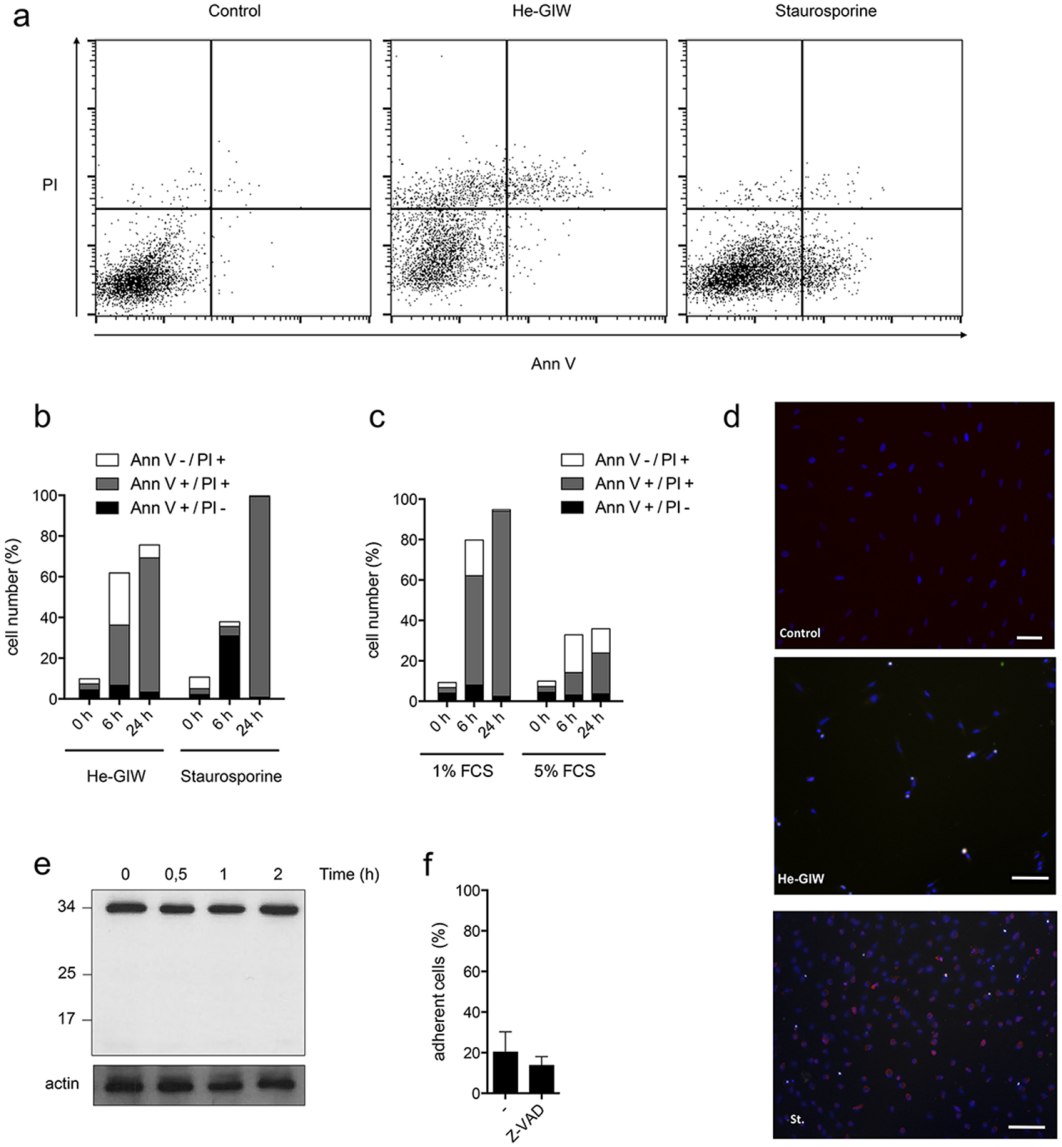
He-GIW induces a necrotic cell death. **(a-c)** Presence of apoptosis was assayed by FACS analysis of Annexin V/PI dual staining. **(a)** An example of flow cytometry profile 6 h after treatment with He-GIW or with staurosporine, a positive control for apoptosis. X axis represents annexin-V-APC staining and Y axis PI staining. **(b)** Percentage of cells positive for Annexin V only (black), PI only (white) or both (grey) was measured 6 h and 24 h after treatment with He-GIW or staurosporine in standard cell culture conditions (c) or with 1% or 5% FCS. **(a-c)** are representative of 3 independent experiments. **(d)** Caspase 3 cleavage (red) was assayed by immunofluorescence 6h after He-GIW or staurosporine treatment. Cell nuclei are labeled with Hoechst and appear blue. **(e)** Caspase 3 cleavage was monitored by Western Blot for 2 h after He-GIW treatment. **(f)** Adherent cell percentage 6 h after He-GIW treatment in presence or absence of caspase inhibitor Z-VAD. Error bars represent S.E.M. of three independent experiments. p<0.05 (*), p<0.01 (**) and p<0.001 (***)

Microscopic observation after He-GIW treatment shows that floating cells exhibit a round, balloon-like, morphology without any membrane blebbing (Fig 4c and Fig a in S1 File) suggesting induction of a cell death, mainly by necrosis. To confirm this observation, cells were stained with annexin V, a biochemical marker of apoptosis, and propidium iodide (PI), a marker of cell membrane integrity. Cell treatment with the prototypical apoptosis inducer, staurosporine, showed the appearance at 6 hours of a cell population that was Annexin V+/PI-. In contrast, He-GIW treatment elicited the appearance at the same time point of a cell population that had taken up PI but was heterogeneous with regard to Annexin V staining (Fig 5a and 5b). To evaluate the integrity of Annexin V+ cells under these conditions, we plotted the forward (FSC) and side (SSC) scatter profile of Annexin V+ cells against the total cell population. Unlike Annexin V+ staurosporine-treated cells, which at 6 hours had a scatter profile consistent with intact cells, Annexin V+ He-GIW-treated cells had an FSC^low^ profile consistent with damaged cells (Fig b in S1 File). This, in combination with the fact that they were also PI+ (Fig 5a and 5b), suggests that these cells may have passively taken up Annexin V due to their damaged state [31]. Later, 24h after treatment with He-GIW or staurosporine, nearly all cells were double positive for Annexin V and PI (Fig 5a and 5b) and damaged (Fig b in S1 File). Although it is difficult to ascertain the exact mechanism of death at this late stage, it is likely, given the absence of Annexin V+/PI- staining early on, that apoptosis was not a major contributor to death of He-GIW-treated cells.

Although increasing serum concentration reduced the dead cell number 24 hrs after He-GIW culture exposure, the early non-apoptotic profile of He-GIW treated cells was not modified (Fig 5c). We confirmed the absence of apoptosis by monitoring the production of cleaved caspase 3 (Fig 5d and 5e) and the lack of inhibition by the pan caspase inhibitor Z-VAD (Fig 5f). These results suggested that He-GIW treatment induces necrosis in hPDL cells treated with He-GIW.

### He-GIW exposure induces a depolarization of the mitochondrial membrane after the first hour and precedes cell death

**Fig 6:**
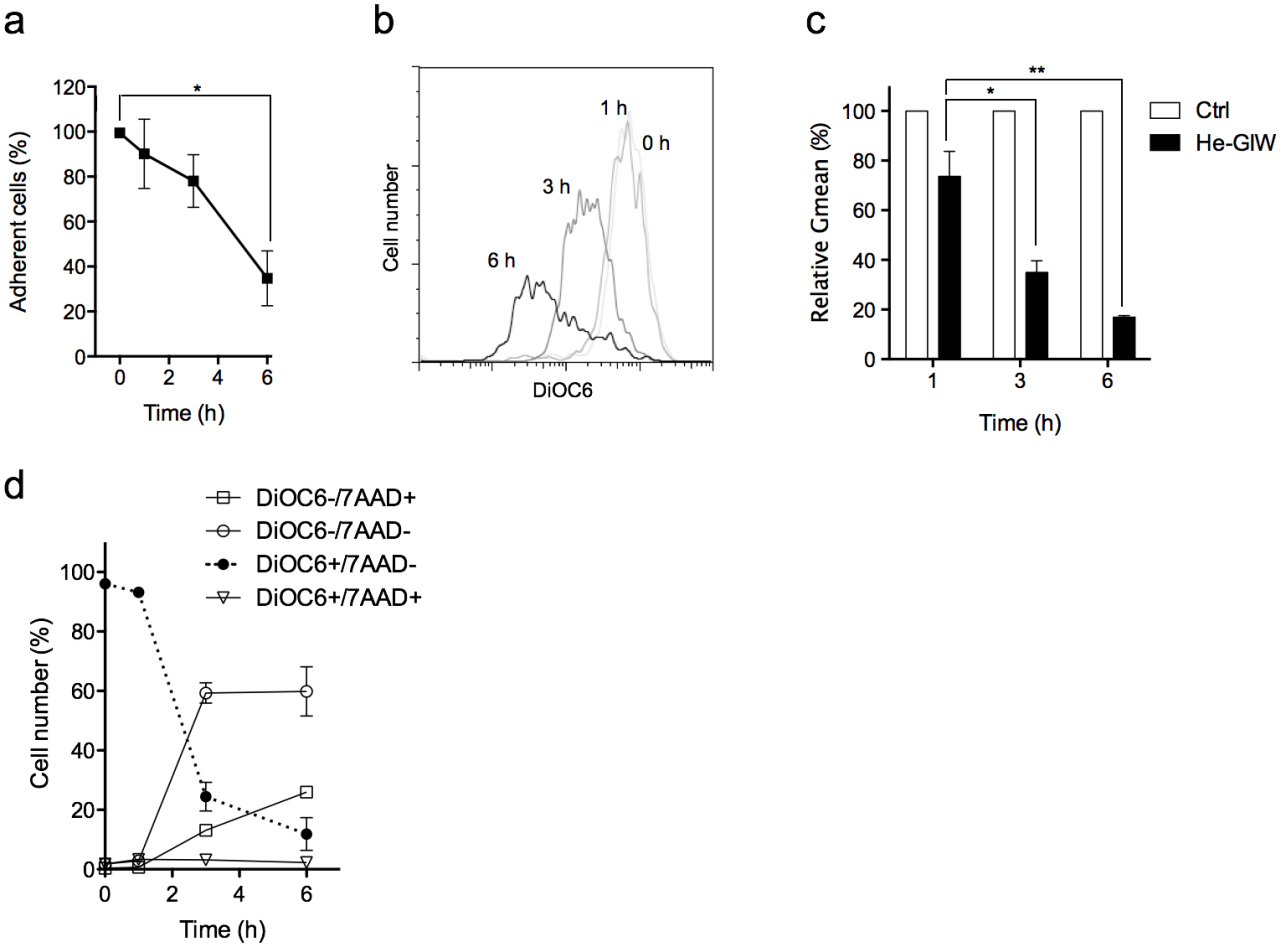
Mitochondrial membrane depolarization precedes cell membrane alteration. **(a)** Adherent cell percentage was monitored for 6 h after He-GIW treatment. **(b)** Cell Δψm was monitored by FACS analysis for 6h using DiOC6 staining. Results of one representative experiment of at least three. **(c)** Geometric means of DiOC6 staining relative to control 1, 3 or 6 h after He-GIW treatment. **(d)** Δψm (DiOC6) and plasma cell membrane permeability (7AAD) were monitored by FACS analysis for 6 h after He-GIW treatment. When present, error bars represent S.E.M. of three independent experiments p<0.05 (*), p<0.01 (**) and p<0.001 (***)

During necrosis, cell membrane permeabilization can be initiated either by direct damage to the plasma cell membrane or by energy depletion. To distinguish between these possibilities, we used DiOC6 marker to monitor mitochondrial inner membrane potential (Δψm), a requirement for ATP production, following He-GIW exposure. Most of He-GIW-treated cells lost adhesion during the first 6 hours (Fig 6a). Δψm remained stable the first hour, whereas 60% of cells lost their Δψm 3 h after treatment (Fig 6b and 6c). DiOC6/7AAD double staining shows that at that time, 60% of the exposed cells that have lost their Δψm remained negative for 7AAD, a marker of cell membrane permeability. This suggests that Δψm impairment precedes the loss of plasma membrane integrity (Fig 6d) and that cell membrane permeabilization observed after He-GIW treatment is due to energetic collapse rather than cell membrane alteration.

### The He-GIW-induced cell toxicity is caused by a modification of the extracellular environment

**Fig 7:**
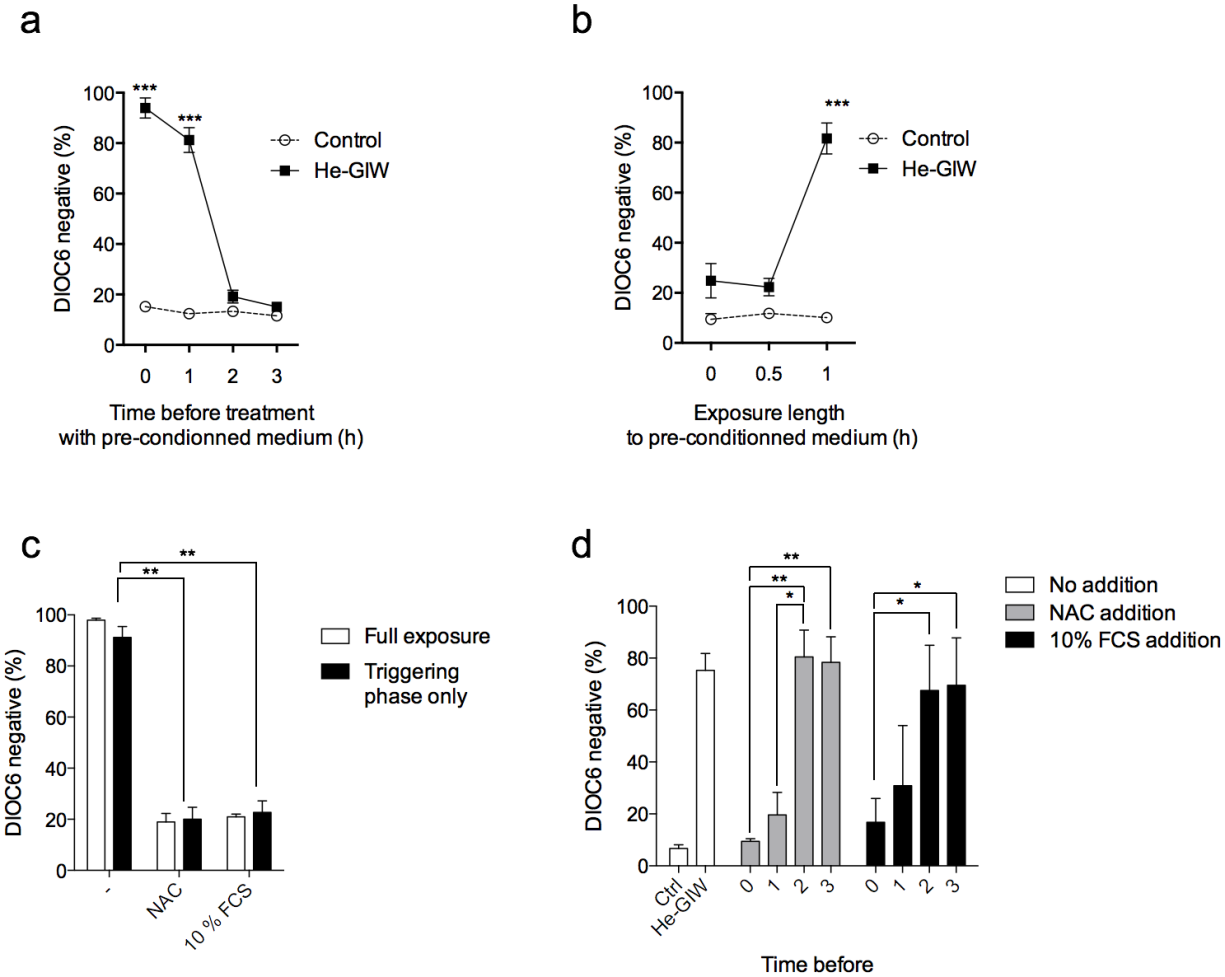
Necrosis induction is indirect and its execution is controlled. **(a)** Cell-free medium was conditioned by treatment with He-GIW. Cells were then treated by conditioned medium immediately, or 1 h, 2 h, or 3 h after He-GIW conditioning. **(b)** Cell medium was renewed before, concomitantly with, or 30 min or 1 h after He-GIW treatment. **(c)** Cells were treated with He-GIW alone or in presence of NAC or 10% FCS. Cell medium was renewed 1h after treatment (Triggering phase only in black) or not (Full exposure in white). **(d)** Cells were treated with He-GIW, and 10% FCS or NAC were added immediately, 1 h, 2h, or 3h after treatment. In every experiment, the percentage of DiOC6 negative cells was assayed 6 h after cell treatment. Note that death can be inhibited even if cells have been exposed to the 1h triggering phase. Error bars represent S.E.M. of three independent experiments. p<0.05 (*), p<0.01 (**) and p<0.001 (***).

We wondered whether alteration of mitochondrial respiration induced by He-GIW was caused by a modification of cell environment or by a direct effect on cells. Cell-free medium was first generated by treatment with He-GIW, then cells were incubated with this conditioned medium. Δψm loss was observed up to 6 h after treatment with cell-free He-GIW-treated medium, suggesting that cell death was induced by interaction of plasma with the medium and not with the cells. Medium alteration was immediate and transient, with the effect of He-GIW treatment on medium persisting up to two hours after conditioning (Fig 7a).

As toxicity was mediated by the extracellular environment, we next determined the minimal time of exposure necessary and sufficient to induce Δψm loss. At various time points after exposure to He-GIW, we replaced cell medium by fresh medium and measured Δψm 3 h later (Fig 7b). Change of culture medium immediately or 30 min after exposure prevented the Δψm impairment, further confirming that Δψm collapse depends on cell medium and not on cell-plasma interaction (Fig 7b). Leaving cells in He-GIW-treated medium for at least 1 h was necessary and sufficient to induce Δψm loss 3 h after treatment, suggesting that cell death was trigged between 30 and 60 minutes after exposure to conditioned extracellular medium (Fig 7b). Collectively, data show that cytotoxicity of He-GIW was caused by a fast and transient change in the cell’s environment. Exposure to this modified medium for as little as 1 h was necessary and sufficient to induce an energetic catastrophe 3 h later. This timeframe is henceforth referred as the triggering phase.

### NAC or 10% FSC addition prevent He-GIW toxicity

Since it has been described that plasma effects can be mediated by ROS, we used the ROS inhibitor N-acetyl cysteine (NAC) in our mitochondrial potential assay. Indeed, addition of NAC at the time of treatment inhibited the Δψm loss induced by He-GIW to the same extent as the addition of 10% FCS (Fig 7c). Protection by NAC or FCS was equally effective, whether these agents were present continuously or only during the triggering phase, thereby confirming their ability to prevent the initiation of cell death (Fig 7c). Surprisingly, protection by NAC, and to a lesser extent by FCS, was also effective if added after the triggering phase, suggesting that both molecules were also able to inhibit cell death execution. However, protection was lost if NAC or FCS were added 2 h after He-GIW, indicating that cells have passed, at that time, beyond a critical control point (Fig 7d).

## Discussion

Heat-producing plasma has historically been used on living tissue only for tissue removal, sterilization, and cauterisation. More recently, Cold Atmospheric Plasma devices have raised interest because they can be tuned to induce selective non-lethal effects at close to room temperatures with a potential use in cancer therapy, wound healing, and tissue regeneration [18]. There is consensus in the field that while the effects of CAP on mammalian cells are mediated by reactive species generation, other energy components of CAP, such as the electric fields, the charges and photons involved in such processes, may play a synergistic role. However, studies aimed at understanding the mechanism of action of plasma using cell culture models have proven difficult to interpret due to pleiotropic effects ranging from disturbing cell adhesion to apoptotic cell death induction. Generally, these controversial data are attributed to differences between devices, settings, or cellular models [22].

Here, we show that hPDL treatment with He-GIW can induce a form of cell death with features distinct from apoptosis. For instance, the caspase proteolytic cascade classically associated with apoptosis was not activated and chemical inhibition of caspases was not able to reverse cell death. Although we cannot rule out the participation of a caspase independent apoptotic pathway, morphological and biochemical observations tend to favour necrosis. Indeed death was accompanied by swelling without membrane blebbing or phosphatidylserine externalization. Therefore, we describe here a non-apoptotic cell death induced by plasma treatment [32–40]. Although this finding may be linked to differences in plasma device or in the cell type used, some key points should be considered.

First, similar results were obtained on connective cells from various origins, like adipose tissues (data not shown). Moreover, the cell death highlighted here is indirect. It relies on one or several transient compounds rapidly generated in the extracellular medium exposed to He-GIW. This observation is in agreement with previous reports [33,36,41,42] showing that cell treatment with plasma depends on their interaction with the cell medium. Moreover, by adding a ROS inhibitor or serum to the medium, we observed that the same device and settings can produce different effects depending on the redox status of the environment. This additional layer of variability should be taken into account when working on plasma/cell interactions *in vitro*.

Little information is available on the mechanisms leading to plasma-induced cell death. Kalghatgi and al. [33] have shown that plasma cell treatment induces the production of intracellular reactive species, leading to DNA single strand breaks and apoptosis 3 days after treatment. Other studies also reported the presence of DNA damage and cell death by apoptosis between 1 and 3 days [40,38,32–37,39] using different cell lines and devices. Panngom and al. [40] recently observed that plasma induces modifications in mitochondria morphology 24 h after treatment, and Kang and al. [43] identified mitochondria as a potent source of intracellular ROS. In our model hPDL treatment by He-GIW predominantly induces a form of necrotic cell death that was due to alteration of mitochondrial function and was effective only 3 h after treatment. Differences in cell death execution and kinetics could reflect quantitative differences in reactive species production by medium, as many insults induce apoptosis at lower doses and necrosis at higher doses [24].

The necrosis we describe in this study is not due to generation of an environment incompatible with hPDL physiology, as it could be inhibited to rescue cells even if they were exposed to potentially lethal doses. For a long time, necrosis has been opposed to apoptosis by only considering it as an accidental and uncontrolled process. However, accumulating evidence shows that execution of necrosis processes could be carried out by signal transduction and inhibited to rescue cells. For example, in cerebral stroke and myocardial infarction, necrosis is not only due to oxygen deprivation as initially thought, but can also be induced by perfusion restoration. While the precise mechanism is still debated, it has been shown that ischemia/reperfusion necrosis can be prevented by p53 inhibition, a signalling pathway involved in stress response, or by ROS scavengers like NAC [24].

Further investigations are needed to determine the key players of the pathway leading to bioenergetics failure in connective cells following He-GIW exposure. While treatment outcome may be specific to a cell type or cell culture condition, this study could open a new rationale to medical applications. We have shown that plasma-induced free radical production depends on the redox status of the environment. Consequently, He-GIW could be used to selectively target cells in permissive context like in tumoral, infected or inflamed tissue [44,45] while preserving normal tissue. For example, periodontitis could represent an interesting target for debridement, as it is particularly prone to oxidative stress [45]. Moreover, since cell death induced by He-GIW is controlled, its execution could be inhibited to preserve fibroblasts and their regenerative potential. This specificity could be also exploited to commit cancer cell to necrosis due to their increased sensitivity to oxidative stress [46,47]. In this case, necrosis is of particular value, as it has been shown that during necrosis cells release molecules that stimulate the immune response, a mechanism that has been correlated to long term chemotherapy success [48]. Understanding the mechanisms underlying cell death in CAP is key to its proper therapeutic use and to prevent eventual undesirable effects.

In summary, we show here that normal primary fibroblasts exposed to a He-GIW CAP undergo non-accidental form of necrosis that is due to an interaction between extracellular environment and plasma rather than to a direct cell response to plasma. This effect may be attributed, at least partly, to specific gaseous species generated by our device, like ozone, as recently suggested [49]. By transiently modifying the cell’s environment, the He-GIW plasma used here induces a cellular response culminating in mitochondrial shutdown. While further studies are needed to pinpoint the nature of the connection between extracellular medium modification and mitochondrial dysfunction, the findings reported here reveal a novel, indirect effect of plasma on cell fate. This new type of effect will need to be taken into account when evaluating the efficiency and safety of plasma treatment.

## Supporting Information

S1 File Cell death morphology differs between HeGIW and staurosporine treatment. Exemple of morphological differences observed by light microscopy between staurosporine and He-GIW treatment at 6 hours (Figure a). Flow cytometry profile showing forward (FSC) and side scattered (SSC) light generated by cells 6 or 24h after treatment with He-GIW or the apoptosis inducer staurosporine. Blue dots represent Annexin V-positive cells and red dots represent the total cell population. Ellipse corresponds to the window that contains the total, untreated cell population at each time point (Figure b).
(TIF)

S2 File Electrical high voltage and current signals analyzed during one impulse (Figure 2a) and during one period (Figure 2b). Relative concentration of the main emissive species in the guided ionization wave (Figure 3). Flow cytometry count for device settings and cell culture parameters (Figure 4). Flow cytometry count for apoptosis assays and apoptosis inhibitor (Figure 5). Flow cytometry count for adherent cell and DiOC6 assays (Figure 6). Flow cytometry count for DiOC6 assays (Figure 7).
(ZIP)

## References

[1] Hong YC, Uhm HS. Microplasma jet at atmospheric pressure. Appl Phys Lett. 2006;89: 221504.

[2] Dudek D, Bibinov N, Engemann J, Awakowicz P. Direct current plasma jet needle source. J Phys Appl Phys. 2007;40: 7367.

[3] Walsh JL, Kong MG. Room-temperature atmospheric argon plasma jet sustained with submicrosecond high-voltage pulses. Appl Phys Lett. 2007;91: 221502.

[4] Weltmann K-D, Brandenburg R, von Woedtke T, Ehlbeck J, Foest R, Stieber M, et al Antimicrobial treatment of heat sensitive products by miniaturized atmospheric pressure plasma jets (APPJs). J Phys Appl Phys. 2008;41: 194008.

[5] Lu X, Jiang Z, Xiong Q, Tang Z, Pan Y. A single electrode room-temperature plasma jet device for biomedical applications. Appl Phys Lett. 2008;92: 151504.

[6] Mericam-Bourdet N, Laroussi M, Begum A, Karakas E. Experimental investigations of plasma bullets. J Phys Appl Phys. 2009;42: 055207.

[7] Jarrige J, Laroussi M, Karakas E. Formation and dynamics of plasma bullets in a non-thermal plasma jet: influence of the high-voltage parameters on the plume characteristics. Plasma Sources Sci Technol. 2010;19: 065005.

[8] Kim SJ, Chung TH, Bae SH, Leem SH. Induction of apoptosis in human breast cancer cells by a pulsed atmospheric pressure plasma jet. Appl Phys Lett. 2010;97: 023702.

[9] Srivastava N, Wang C. Determination of OH Radicals in an Atmospheric Pressure Helium Microwave Plasma Jet. IEEE Trans Plasma Sci. 2011;39: 918–924. 10.1109/TPS.2010.2101618

[10] Joh HM, Kim SJ, Chung TH, Leem SH. Reactive oxygen species-related plasma effects on the apoptosis of human bladder cancer cells in atmospheric pressure pulsed plasma jets. Appl Phys Lett. 2012;101: 053703.

[11] Reuter S, Winter J, Iseni S, Peters S, Schmidt-Bleker A, Dünnbier M, et al Detection of ozone in a MHz argon plasma bullet jet. Plasma Sources Sci Technol. 2012;21: 034015.

[12] van Gils CAJ, Hofmann S, Boekema BKHL, Brandenburg R, Bruggeman PJ. Mechanisms of bacterial inactivation in the liquid phase induced by a remote RF cold atmospheric pressure plasma jet. J Phys Appl Phys. 2013;46: 175203.

[13] Xiong Q, Nikiforov AY, González MÁ, Leys C, Lu XP. Characterization of an atmospheric helium plasma jet by relative and absolute optical emission spectroscopy. Plasma Sources Sci Technol. 2013;22: 015011.

[14] Robert E, Sarron V, Riès D, Dozias S, Vandamme M, Pouvesle J-M. Characterization of pulsed atmospheric-pressure plasma streams (PAPS) generated by a plasma gun. Plasma Sources Sci Technol. 2012;21: 034017.

[15] Boeuf J-P, Yang LL, Pitchford LC. Dynamics of a guided streamer (‘plasma bullet’) in a helium jet in air at atmospheric pressure. J Phys Appl Phys. 2013;46: 015201.

[16] Gazeli K, Noël C, Clément F, Daugé C, Svarnas P, Belmonte T. A study of helium atmospheric-pressure guided streamers for potential biological applications. Plasma Sources Sci Technol. 2013;22: 025020.

[17] Lu X, Naidis GV, Laroussi M, Ostrikov K. Guided ionization waves: Theory and experiments. Phys Rep. 2014;540: 123–166. 10.1016/j.physrep.2014.02.006

[18] Fridman G, Friedman G, Gutsol A, Shekhter AB, Vasilets VN, Fridman A. Applied Plasma Medicine. Plasma Process Polym. 2008;5: 503–533. 10.1002/ppap.200700154

[19] Kémoun P, Gronthos S, Snead ML, Rue J, Courtois B, Vaysse F, et al The role of cell surface markers and enamel matrix derivatives on human periodontal ligament mesenchymal progenitor responses in vitro. Biomaterials. 2011;32: 7375–7388. 10.1016/j.biomaterials.2011.06.043 21784516 PMC4441221

[20] Foster B, Somerman M. Regenerating the periodontium: is there a magic formula? Orthod Craniofac Res. 2005;8: 285–291. 10.1111/j.1601-6343.2005.00351.x 16238609

[21] Aichelmann-Reidy ME, Reynolds MA. Predictability of Clinical Outcomes Following Regenerative Therapy in Intrabony Defects. J Periodontol. 2008;79: 387–393. 10.1902/jop.2008.060521 18315419

[22] Dobrynin D, Fridman G, Friedman G, Fridman A. Physical and biological mechanisms of direct plasma interaction with living tissue. New J Phys. 2009;11: 115020.

[23] Lockshin RA, Zakeri Z. Programmed cell death and apoptosis: origins of the theory. Nat Rev Mol Cell Biol. 2001;2: 545–550. 11433369 10.1038/35080097

[24] Zong W-X, Thompson CB. Necrotic death as a cell fate. Genes Dev. 2006;20: 1–15. 10.1101/gad.1376506 16391229

[25] Berghe TV, Linkermann A, Jouan-Lanhouet S, Walczak H, Vandenabeele P. Regulated necrosis: the expanding network of non-apoptotic cell death pathways. Nat Rev Mol Cell Biol. 2014;15: 135–147. 10.1038/nrm3737 24452471

[26] Galluzzi L, Vitale I, Abrams JM, Alnemri ES, Baehrecke EH, Blagosklonny MV, et al Molecular definitions of cell death subroutines: recommendations of the Nomenclature Committee on Cell Death 2012. Cell Death Differ. 2012;19: 107–120. 10.1038/cdd.2011.96 21760595 PMC3252826

[27] Gazeli K., Clément F., Le T.D., Svarnas P., Duday D., Daugé C., Held B., Investigation of electrical and thermal characteristics of DBD-based plasma jet at atmospheric pressure, XIX International Conference on Gas Discharges and their applications (GD2012), Beijing, China, vol 38, 2012

[28] Gazeli K, Svarnas P, Held B, Marlin L, Clément F. Possibility of controlling the chemical pattern of He and Ar “guided streamers” by means of N2 or O2 additives. J Appl Phys. 2015;117: 093302.

[29] Laroussi M, Lu X, Kolobov V, Arslanbekov R. Power consideration in the pulsed dielectric barrier discharge at atmospheric pressure. J Appl Phys. 2004;96: 3028–3030.

[30] Marchetti C, Obert G, Deffosez A, Formstecher P, Marchetti P. Study of mitochondrial membrane potential, reactive oxygen species, DNA fragmentation and cell viability by flow cytometry in human sperm. Hum Reprod Oxf Engl. 2002;17: 1257–1265. 10.1093/humrep/17.5.1257 11980749

[31] Krysko DV, Vanden Berghe T, D’Herde K, Vandenabeele P. Apoptosis and necrosis: Detection, discrimination and phagocytosis. Methods San Diego Calif. 2008;44: 205–21. 10.1016/j.ymeth.2007.12.001 18314051

[32] Fridman G, Shereshevsky A, Jost MM, Brooks AD, Fridman A, Gutsol A, et al Floating Electrode Dielectric Barrier Discharge Plasma in Air Promoting Apoptotic Behavior in Melanoma Skin Cancer Cell Lines. Plasma Chem Plasma Process. 2007;27: 163–176. 10.1007/s11090-007-9048-4

[33] Kalghatgi S, Kelly CM, Cerchar E, Torabi B, Alekseev O, Fridman A, et al Effects of Non-Thermal Plasma on Mammalian Cells. PLoS ONE. 2011;6: e16270 10.1371/journal.pone.0016270 21283714 PMC3025030

[34] Kieft IE, Kurdi M, Stoffels E. Reattachment and Apoptosis After Plasma-Needle Treatment of Cultured Cells. IEEE Trans Plasma Sci. 2006;34: 1331–1336. 10.1109/TPS.2006.876511

[35] Lee HJ, Shon CH, Kim YS, Kim S, Kim GC, Kong MG. Degradation of adhesion molecules of G361 melanoma cells by a non-thermal atmospheric pressure microplasma. New J Phys. 2009;11: 115026.

[36] Ryu Y-H, Kim Y-H, Lee J-Y, Shim G-B, Uhm H-S, Park G, et al Effects of Background Fluid on the Efficiency of Inactivating Yeast with Non-Thermal Atmospheric Pressure Plasma. PLoS ONE. 2013;8: e66231 10.1371/journal.pone.0066231 23799081 PMC3683031

[37] Stoffels E, Kieft IE, Sladek REJ. Superficial treatment of mammalian cells using plasma needle. J Phys Appl Phys. 2003;36: 2908.

[38] Chang JW, Kang SU, Shin YS, Kim KI, Seo SJ, Yang SS, et al Non-thermal atmospheric pressure plasma induces apoptosis in oral cavity squamous cell carcinoma: Involvement of DNA-damage-triggering sub-G1 arrest via the ATM/p53 pathway. Arch Biochem Biophys. 2014;545: 133–140. 10.1016/j.abb.2014.01.022 24486404

[39] Ma Y, Ha CS, Hwang SW, Lee HJ, Kim GC, Lee K-W, et al Non-Thermal Atmospheric Pressure Plasma Preferentially Induces Apoptosis in p53-Mutated Cancer Cells by Activating ROS Stress-Response Pathways. PLoS ONE. 2014;9: e91947 10.1371/journal.pone.0091947 24759730 PMC3997341

[40] Panngom K, Baik KY, Nam MK, Han JH, Rhim H, Choi EH. Preferential killing of human lung cancer cell lines with mitochondrial dysfunction by nonthermal dielectric barrier discharge plasma. Cell Death Dis. 2013;4: e642 10.1038/cddis.2013.168 23703387 PMC3674375

[41] Vandamme M, Robert E, Lerondel S, Sarron V, Ries D, Dozias S, et al ROS implication in a new antitumor strategy based on non-thermal plasma. Int J Cancer J Int Cancer. 2012;130: 2185–2194. 10.1002/ijc.26252 21702038

[42] Ahn HJ, Kim KI, Hoan NN, Kim CH, Moon E, Choi KS, et al Targeting Cancer Cells with Reactive Oxygen and Nitrogen Species Generated by Atmospheric-Pressure Air Plasma. PLoS ONE. 2014;9: e86173 10.1371/journal.pone.0086173 24465942 PMC3897664

[43] Kang SU, Cho J-H, Chang JW, Shin YS, Kim KI, Park JK, et al Nonthermal plasma induces head and neck cancer cell death: the potential involvement of mitogen-activated protein kinase-dependent mitochondrial reactive oxygen species. Cell Death Dis. 2014;5: e1056 10.1038/cddis.2014.33 24525732 PMC3944250

[44] Schafer FQ, Buettner GR. Redox environment of the cell as viewed through the redox state of the glutathione disulfide/glutathione couple. Free Radic Biol Med. 2001;30: 1191–1212. 10.1016/S0891-5849(01)00480-4 11368918

[45] Chapple ILC, Matthews JB. The role of reactive oxygen and antioxidant species in periodontal tissue destruction. Periodontol 2000. 2007;43: 160–232. 10.1111/j.1600-0757.2006.00178.x 17214840

[46] Trachootham D, Alexandre J, Huang P. Targeting cancer cells by ROS-mediated mechanisms: a radical therapeutic approach? Nat Rev Drug Discov. 2009;8: 579–591. 10.1038/nrd2803 19478820

[47] Bedner E, Du L, Traganos F, Darzynkiewicz Z. Caffeine dissociates complexes between DNA and intercalating dyes: application for bleaching fluorochrome-stained cells for their subsequent restaining and analysis by laser scanning cytometry. Cytometry. 2001;43: 38–45. 11122483

[48] Kroemer G, Galluzzi L, Kepp O, Zitvogel L. Immunogenic Cell Death in Cancer Therapy. Annu Rev Immunol. 2013;31: 51–72. 10.1146/annurev-immunol-032712-100008 23157435

[49] Lunov O, Zablotskii V, Churpita O, Chánová E, Syková E, Dejneka A, et al Cell death induced by ozone and various non-thermal plasmas: therapeutic perspectives and limitations. Sci Rep. 2014;4 10.1038/srep07129 PMC4238021 25410636

