# Supplementary figures and images for: Cold Atmospheric Plasma Induces a Predominantly Necrotic Cell Death via the Microenvironment
Source: PLoS One. 2015 Aug 14;10(8):e0133120. doi: 10.1371/journal.pone.0133120 (PMC4537210; doi:10.1371/journal.pone.0133120)

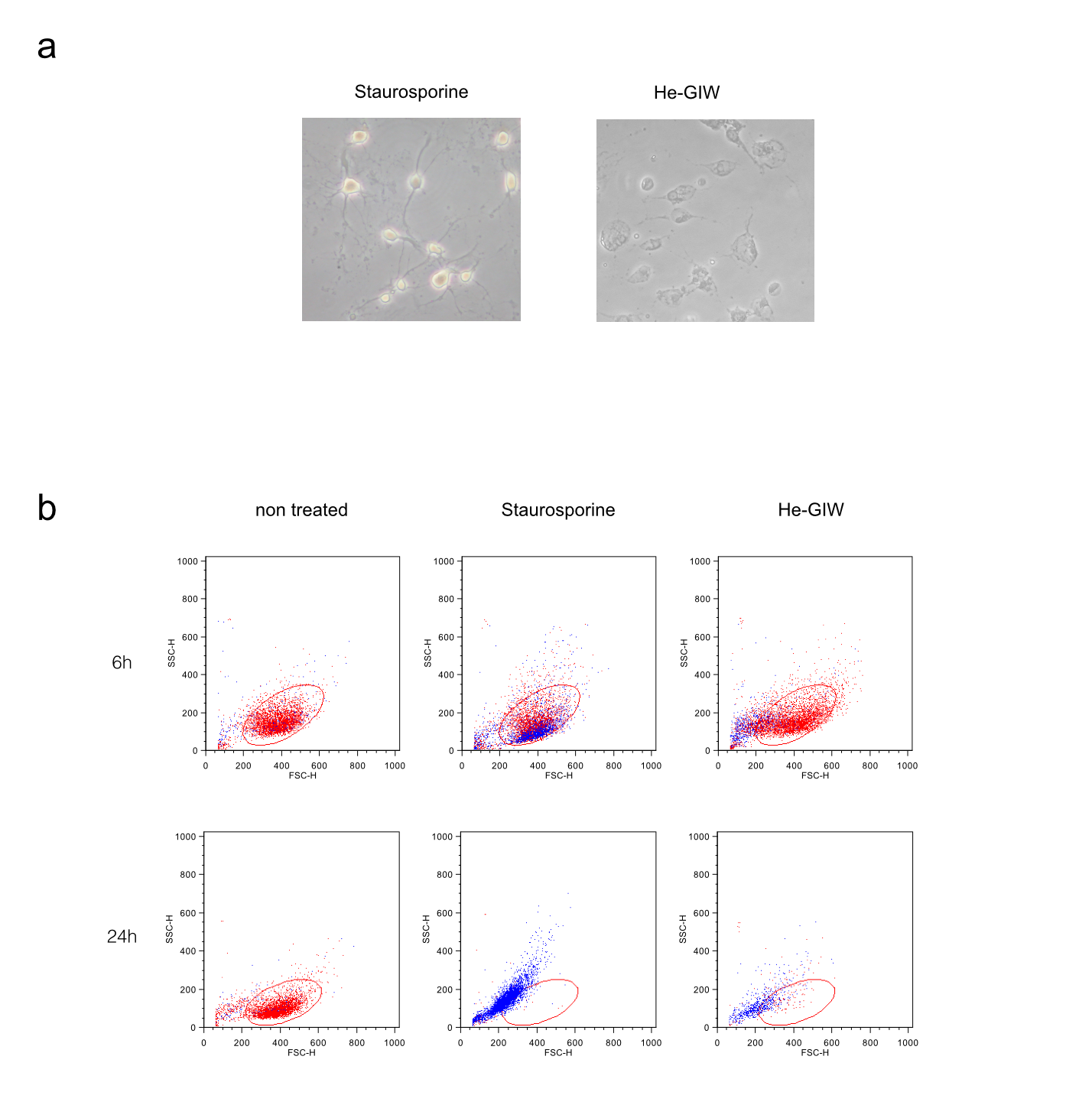

Supplement: S1 File — Exemple of morphological differences observed by light microscopy between staurosporine and He-GIW treatment at 6 hours (Figure a). Flow cytometry profile showing forward (FSC) and side scattered (SSC) light generated by cells 6 or 24h after treatment with He-GIW or the apoptosis inducer staurosporine. Blue dots represent Annexin V-positive cells and red dots represent the total cell population. Ellipse corresponds to the window that contains the total, untreated cell population at each time point (Figure b). (TIF) [file pone.0133120.s001.tif]
